# Outcomes with venoarterial vs venovenous extracorporeal membrane oxygenation as a bridge to lung transplantation in interstitial lung disease

**DOI:** 10.1016/j.jhlto.2025.100287

**Published:** 2025-05-13

**Authors:** Prasanth Balasubramanian, Pablo Moreno Franco, Sanjay Chaudhary, Francisco G. Alvarez, Maher Baz, Tathagat Narula, Sadia Z. Shah, Mohammad Alomari, Rohan Bongu, Anirban Bhattacharrya, Devang Sanghavi, Sean Kiley, Archer K. Martin, Nikki L. Matos, John C. Haney, Ian Makey, Mathew Thomas, Pramod K. Guru, Remzi Bag

**Affiliations:** a Division of Lung Failure and Transplant, Department of Transplantation, Mayo Clinic, Jacksonville, Florida; b Department of Cardiothoracic Surgery, Mayo Clinic, Jacksonville, Florida; c Department of Critical Care Medicine, Mayo Clinic, Jacksonville, Florida; d Division of Cardiovascular and Thoracic Anesthesiology, Department of Anesthesia and Peri-op Medicine, Mayo Clinic, Jacksonville, Florida

**Keywords:** lung transplantation, interstitial lung disease (ILD), extracorporeal membrane oxygenation (ECMO), pulmonary hypertension, cannulation

## Abstract

**Background:**

Evidence on outcomes associated with venoarterial (VA) versus venovenous (VV) of extracorporeal membrane oxygenation (ECMO) as a bridge to lung transplantation (LTx) in interstitial lung disease (ILD) is limited.

**Methods:**

Using the United Network for Organ Sharing (UNOS) Standard Transplant Analysis and Research (STAR) registry, we conducted a retrospective cohort study on ILD patients bridged with ECMO for LTx. Outcomes by cannulation type (VV vs VA) were analyzed, with 1:1 propensity matching to reduce selection bias.

**Results:**

Among 1,551 patients, 1,236 (79.7%) were on VV ECMO and 315 (20.3%) on VA ECMO. Delisting due to death or deterioration was higher with VA ECMO in both the overall (41% vs 27%, *p* < 0.001) and matched (41% vs 30%, *p* = 0.003) cohorts. VA ECMO was associated with higher 1-year post-transplant mortality in the overall cohort (33% vs 24%, *p* < 0.001), though not statistically significant in the matched cohort (33% vs 24%, *p* = 0.059). Multivariate Cox-proportional hazard regression analysis showed no significant difference in 1-year post-transplant mortality with VA ECMO in the overall (adjusted Hazard Ratio (aHR) 1.01 (95% confidence interval, CI 0.65-1.60, *p* > 0.9)) and matched (aHR 1.24, 95% CI 0.78-1.97, *p* = 0.4) cohorts, compared to VV ECMO. However, the VA ECMO had higher 1-year post-transplant mortality in the setting of COVID-19 associated pulmonary fibrosis (aHR 7.12, 95% CI 1.9-26.6, *p* = 0.003).

**Conclusions:**

VV ECMO was associated with a more successful bridge to LTx than VA ECMO. Adjusted post-transplant outcomes were comparable. Prospective studies are warranted to explore outcome differences by cannulation strategy in ILD.

## Background

Lung transplantation (LTx) is the only therapeutic option that improves the survival and functional status of patients with end-stage lung disease (ESLD). The severe shortage of donor organs and an increasing number of acutely ill candidates have put prospective recipients at the risk of longer wait times, increasing the risk of clinical deterioration and waitlist mortality. Extracorporeal life support via extracorporeal membrane oxygenation (ECMO) has been increasingly used as a bridge to LTx as it allows physical rehabilitation for reconditioning and maintains eligibility for LTx candidacy.^1^, ^2^

ECMO support in ESLD could be provided via venovenous (VV) or venoarterial (VA) configuration. VV ECMO helps with oxygenation and decarboxylation and is the commonly used configuration in the setting of respiratory failure.^3^ In addition to respiratory support, VA ECMO can also provide cardiac support, especially during right ventricular (RV) failure due to decompensated pulmonary hypertension (PH), which is commonly encountered in ESLD. The VA ECMO, however, is associated with specific complications including bleeding, limb ischemia, infection, hemolysis, neurological complications, and north-south syndrome.^4^, ^5^, ^6^

Among the ESLD, interstitial lung disease (ILD) is the most common etiology bridged with ECMO for LTx.^7^ Prevalence of pulmonary hypertension associated with ILD (PH-ILD) is thought to increase with progressive ILD and during acute exacerbations due to a combination of chronic hypoxia, endothelial dysfunction, and vascular remodeling manifest with reduced functional vascular bed and can be seen in up to 80% of the patients.^8^ Increasing pulmonary vascular resistance (PVR) associated with progressive PH accompanying worsening ILD tasks the RV to generate higher pressures to maintain pulmonary circulation, leading to RV dysfunction. Fluid shifts and hemodynamic changes compound RV dysfunction and may lead to RV failure. The RV dysfunction may also worsen during increased preload and higher cardiac output (CO) demand during ambulation while on ECMO. Thus, VA ECMO could have theoretical advantages over VV ECMO in the setting of PH-ILD with concomitant RV dysfunction/failure. However, prior studies have shown that up to two-thirds of patients with ILD are bridged to LTx with VV ECMO.^7^

Data on outcomes of VA vs VV ECMO in the setting of ILD are limited. A small retrospective study on ILD patients with PH-ILD showed that patients bridged with VA ECMO had higher likelihood of successful LTx.^9^ A meta-analysis showed similar trends in terms of improved survival in patients with ILD bridged with VA ECMO compared to VV ECMO among those who are ambulatory, with a “very-low Grading of Recommendations Assessment, Development and Evaluation (GRADE) certainty” of the findings.^10^ However, there have been no further studies to evaluate the outcomes of VA vs VV ECMO in the setting of ILD with ECMO as a bridge to transplant, specifically with a larger sample size.

## Methods

### Study design

We conducted a retrospective cohort analysis of patients with ILD (United Network for Organ Sharing [UNOS] Group D) listed for LTx while on ECMO at the time of listing using the UNOS Organ Procurement and Transplantation Network's Standard Transplant Analysis and Research (STAR) registry. Data were excluded if ECMO was initiated during or after LTx. The timeframe of the data was between September 17, 2010 (the first available data with ECMO in the registry) and March 30, 2024. This study was approved by the institutional review board at the Mayo Clinic (IRB #24-004908).

### Measurements and outcomes

We collected data on baseline demographics, pulmonary diagnosis, bilirubin, creatinine, and 6-minute walk distance. Invasive hemodynamic data at listing, including central venous pressure (CVP), pulmonary artery pressures (PAP), pulmonary capillary wedge pressure (PCWP), and CO, were also gathered. Additional variables included inotrope or pressor use, ambulatory status, ECMO configuration changes, cannulation approach (central vs peripheral), and listing type (single vs bilateral LTx). Lung allocation score (LAS) data were collected until March 9, 2023, and composite allocation score (CAS) thereafter. Pulmonary vascular resistance (PVR) was calculated as PVR = (mean PAP − PCWP)/CO, and pulmonary artery pulsatility index (PAPI) was calculated as PAPI = (Systolic PAP − Diastolic PAP)/CVP.^11^ The primary predictor variable was the type of ECMO at listing (VA or VV ECMO). The primary outcome was 1-year post-transplant mortality. Secondary outcomes included delisting due to deterioration or death, mortality at 30 days, 90 days, and 5 years post-transplant, and the need for ventilator or ECMO support 72 hours post-transplant.

### Statistical analysis

Continuous variables were reported as mean ± standard deviation or median with interquartile range, based on data normality, and compared between groups using Welch’s *t*-test for means and the Wilcoxon rank-sum test for medians. Categorical variables were compared using Pearson’s chi-square or Fisher’s exact test, depending on cell counts. To balance baseline demographics, disease severity (based on LAS or CAS), laboratory values, and invasive hemodynamics between VA and VV ECMO patients, 1:1 propensity score matching was conducted using the nearest neighbor algorithm, achieving an absolute standardized mean difference <0.2 (Figure S1).

The primary outcome of 1-year post-transplant mortality was assessed using Cox-proportional hazard multivariate regression analysis, adjusted for relevant variables identified via directed acyclic graph: age, sex, diabetes, blood group, White race, body mass index (BMI), idiopathic pulmonary fibrosis (IPF) diagnosis, LAS, creatinine, bilirubin, 6-minute walk distance, PVR, use of inotropes, inhaled nitric oxide, prostacyclin infusion, ECMO cannulation type at listing, days from admission to ECMO initiation, cannulation approach (central vs peripheral), ambulatory status, and listing for bilateral LTx (Figure S2). Missing data were imputed using multiple imputation by chained equations for multivariate regression and generalized additive models; missing LAS values post March 9, 2023 (due to CAS replacement) were also imputed. Missing outcome data were not imputed, and such cases were excluded from the multivariate analysis. Subgroup analyses were performed by age, ILD type, ambulatory status, PVR, year of transplant, ECMO cannulation approach, and with the unimputed dataset. The survival until 5 years post-transplant was analyzed using Kaplan-Meier survival curves, and the log-rank test was used to assess the statistical significance. Statistical analyses were conducted using R 4.2.3 with “gtsummary,” “mgcv,” and “ggplot2” packages.

## Results

The cohort comprised 1,551 ILD patients, with 315 (20.3%) listed on VA ECMO and 1,236 (79.7%) on VV ECMO. The mean age was 51 ± 14 years; 62% were male, and 60% were Caucasian. The primary ILD etiology was IPF (37%), followed by COVID-related fibrosis (16%) and connective tissue disease–related ILD (CTD-ILD) (7.2%). Patients on VA ECMO had a significantly higher proportion of Black race (20% vs 11%), CTD-ILD (13% vs 5.7%), systolic PAP (54 vs 44 mm Hg), mean PAP (34 vs 28 mm Hg), PCWP (10.7 vs 9.8 mm Hg), calculated PVR (5.46 vs 3.53), and lower CO (5.16 vs 5.88 liter/min) compared to those on VV ECMO, all of which were statistically significant. Inotrope and pulmonary vasodilator use was similar between groups. Days from admission to ECMO initiation (9 days for both) and ambulatory status on ECMO (36% vs 41%, *p* = 0.13) were also comparable. In the matched cohort of 315 patients per group, baseline demographics were balanced, except for higher COVID–related pulmonary fibrosis in VV ECMO patients (11% vs 7.6%) and higher CTD-ILD in VA ECMO patients (13% vs 7%, *p* = 0.033). Additional details are provided in Table 1.

**Table 1:** Baseline Descriptives of the Overall and Matched Cohorts of Patients With Interstitial Lung Disease Listed for Lung Transplantation While Being on Extracorporeal Membrane Oxygenation

	Overall cohort					Matched cohort				
Characteristic	N	Overall N = 1,551^a^	ECMO VA N = 315^a^	ECMO VV N = 1,236^a^	*p*-value	N	Overall N = 630^a^	ECMO VA N = 315^a^	ECMO VV N = 315^a^	*p*-value
Age (years)	1,551	51 (14)	51 (13)	51 (14)	0.9^b^	630	51 (14)	51 (13)	51 (15)	0.7^b^
Gender	1,551				0.13^c^	630				0.7^c^
Female		592 (38%)	132 (42%)	460 (37%)			269 (43%)	132 (42%)	137 (43%)	
Male		959 (62%)	183 (58%)	776 (63%)			361 (57%)	183 (58%)	178 (57%)	
Blood group	1,551				0.9^c^	630				0.6^c^
A		498 (32%)	98 (31%)	400 (32%)			195 (31%)	98 (31%)	97 (31%)	
AB		62 (4.0%)	11 (3.5%)	51 (4.1%)			29 (4.6%)	11 (3.5%)	18 (5.7%)	
B		187 (12%)	41 (13%)	146 (12%)			78 (12%)	41 (13%)	37 (12%)	
O		804 (52%)	165 (52%)	639 (52%)			328 (52%)	165 (52%)	163 (52%)	
Ethnicity	1,551				**<0.001**^d^	630				0.2^d^
Asian		87 (5.6%)	17 (5.4%)	70 (5.7%)			40 (6.3%)	17 (5.4%)	23 (7.3%)	
Black		193 (12%)	62 (20%)	131 (11%)			107 (17%)	62 (20%)	45 (14%)	
Hispanic/Latino		326 (21%)	61 (19%)	265 (21%)			132 (21%)	61 (19%)	71 (23%)	
Others		22 (1.4%)	1 (0.3%)	21 (1.7%)			5 (0.8%)	1 (0.3%)	4 (1.3%)	
White		923 (60%)	174 (55%)	749 (61%)			346 (55%)	174 (55%)	172 (55%)	
Height	1,551	169 (14)	168 (10)	169 (14)	0.2^b^	630	167 (16)	168 (10)	166 (20)	0.13^b^
BMI (kg/m^b^)	1,538	27.1 (8.0)	27.5 (10.4)	27.0 (7.3)	0.4^b^	626	27.1 (8.3)	27.5 (10.4)	26.7 (5.6)	0.3^b^
Diagnosis	1,551				**<0.001**^c^	630				**0.033**^c^
COVID pulmonary fibrosis		249 (16%)	24 (7.6%)	225 (18%)			59 (9.4%)	24 (7.6%)	35 (11%)	
CTD-ILD		112 (7.2%)	42 (13%)	70 (5.7%)			64 (10%)	42 (13%)	22 (7.0%)	
IPF		580 (37%)	116 (37%)	464 (38%)			242 (38%)	116 (37%)	126 (40%)	
Others		610 (39%)	133 (42%)	477 (39%)			265 (42%)	133 (42%)	132 (42%)	
LAS at listing*	1,368	72 (22)	69 (21)	73 (22)	**0.005**^b^	562	69 (22)	69 (21)	69 (23)	0.8^b^
CAS at listing	183	66 (18)	69 (19)	65 (18)	0.4^b^	68	66 (17)	69 (19)	64 (17)	0.3^b^
6-Minute walk distance at registration	1,506	69 (0, 532)	245 (0, 656)	30 (0, 500)	**<0.001**^e^	608	239 (0, 670)	245 (0, 656)	225 (0, 700)	0.8^e^
Diabetes	1,536	389 (25%)	73 (23%)	316 (26%)	0.3^c^	622	163 (26%)	73 (23%)	90 (29%)	0.090^c^
Initial CVP	1,254	5.0 (2.0, 8.0)	5.0 (2.0, 9.0)	5.0 (2.0, 8.0)	0.2^e^	534	5.0 (2.0, 9.0)	5.0 (2.0, 9.0)	5.0 (2.5, 9.0)	0.3^e^
PA systolic at registration (mm Hg)	1,266	46 (18)	54 (20)	44 (17)	**<0.001**^b^	548	53 (20)	54 (20)	52 (21)	0.2^b^
PA mean at registration (mm Hg)	1,256	29 (12)	34 (14)	28 (12)	**<0.001**^b^	541	34 (14)	34 (14)	34 (15)	0.6^b^
PCWP at registration (mm Hg)	1,228	10.0 (6.2)	10.7 (6.4)	9.8 (6.1)	**0.027**^b^	525	11 (7)	11 (6)	11 (7)	0.4^b^
Cardiac output at registration (liter/min)	1,200	5.72 (2.22)	5.16 (1.85)	5.88 (2.29)	**<0.001**^b^	522	5.14 (1.75)	5.16 (1.85)	5.12 (1.64)	0.8^b^
Calculated PVR at registration (Woods unit)	1,160	3.96 (4.03)	5.46 (4.60)	3.53 (3.75)	**<0.001**^b^	498	5.4 (5.4)	5.5 (4.6)	5.2 (6.2)	0.7^b^
Calculated PAPI	1,136	5 (3, 10)	5 (3, 11)	5 (3, 9)	0.051^e^	496	5 (3, 10)	5 (3, 11)	5 (3, 9)	**0.029**^e^
Inotropes at registration	1,199	88 (7.3%)	20 (7.4%)	68 (7.3%)	>0.9^c^	522	40 (7.7%)	20 (7.4%)	20 (7.9%)	0.8^c^
Inhaled NO at registration	1,053	41 (3.9%)	9 (5.0%)	32 (3.7%)	0.4^c^	390	23 (5.9%)	9 (5.0%)	14 (6.6%)	0.5^c^
Prostacyclin infusion at registration	1,551	12 (0.8%)	2 (0.6%)	10 (0.8%)	>0.9^d^	630	7 (1.1%)	2 (0.6%)	5 (1.6%)	0.5^d^
Bilirubin at registration (mg/dl)	1,544	0.60 (0.40, 0.90)	0.60 (0.40, 1.00)	0.50 (0.40, 0.80)	0.13^b^	624	0.60 (0.40, 0.90)	0.60 (0.40, 1.00)	0.60 (0.40, 0.80)	0.14^b^
Creatinine at registration (mg/dl)	1,549	0.70 (0.52, 0.87)	0.73 (0.59, 0.91)	0.68 (0.51, 0.86)	**0.002**^b^	628	0.73 (0.58, 0.91)	0.73 (0.59, 0.91)	0.72 (0.56, 0.91)	0.9^b^
Central ECMO cannulation	1,550	683 (44%)	178 (57%)	505 (41%)	**<0.001**^c^	630	359 (57%)	178 (57%)	181 (57%)	0.8^c^
Days from admission to ECMO initiation	812	9 (3, 20)	9 (3, 19)	9 (3, 20)	0.7^e^	323	10 (4, 19)	9 (3, 19)	10 (5, 21)	0.13^e^
Ambulatory status	1,551	614 (40%)	113 (36%)	501 (41%)	0.13^c^	630	232 (37%)	113 (36%)	119 (38%)	0.6^c^
VA to VV conversion	1,551	12 (0.8%)	12 (3.8%)	NA	NA	630	12 (1.9%)	12 (3.8%)	NA	NA
VV to VA conversion	1,551	51 (3.3%)	NA	51 (4.1%)	NA	630	20 (3.2%)	NA	20 (6.3%)	NA
Listed for bilateral transplant	1,551	1,470 (95%)	298 (95%)	1,172 (95%)	0.9^c^	630	592 (94%)	298 (95%)	294 (93%)	0.5^c^

Abbreviations: BMI, body mass index; CAS, composite allocation score; CTD-ILD, connective tissue disease–related interstitial lung disease; CVP, central venous pressure; ECMO, extracorporeal membrane oxygenation; IPF, idiopathic pulmonary fibrosis; LAS, lung allocation score; NO, nitric oxide; PA, pulmonary artery; PAPI, pulmonary artery pulsatility index; PCWP, pulmonary capillary wedge pressure; PVR, pulmonary vascular resistance; VA, venoarterial; VV, venovenous.

Bold value represents *p*<0.05.

a Continuous variables—mean (SD) or median (Q1, Q3), categorical variables—frequency (%).

b Welch two sample *t*-test.

c Pearson's chi-square test.

d Fisher's exact test.

e Wilcoxon rank-sum test.

### Outcomes

Of the cohort, 1,053 patients (67.8%) received LTx, 459 (30%) were delisted due to death or deterioration, and 39 (2.5%) were delisted due to clinical improvement. The primary outcome of 1-year post-transplant mortality was higher for patients on VA compared with VV ECMO, but was of statistical significance only in the overall (33% vs 24%, *p* = 0.014), and not in the matched cohort (33% vs 24%, *p* = 0.059). Post-transplant mortality in the overall cohort for VA vs VV ECMO was as follows: 30 days (10% vs 7%, *p* = 0.14), 90 days (18% vs 13%, *p* = 0.065), and 5 years (56% vs 65%, *p* = 0.2). In the matched cohort, mortality at 30 days, 90 days, and 5 years was 10% vs 9.4%, 18% vs 14%, and 56% vs 67%, with none of them meeting statistical significance. Bridging with VA ECMO resulted in a significantly lower transplant rate compared to VV ECMO in both the overall (57% vs 71%, *p* < 0.001) and matched (57% vs 67%, *p* = 0.009) cohorts (Table 2).

**Table 2:** Outcomes Among the Unmatched and Matched Cohorts of Patients With Interstitial Lung Disease Listed for Lung Transplantation While Being on Extracorporeal Membrane Oxygenation

	Overall cohort					Matched cohort				
Characteristic	N	Overall N = 1,551^a^	ECMO VA N = 315^a^	ECMO VV N = 1,236^a^	*p*-value	N	Overall N = 630^a^	ECMO VA N = 315^a^	ECMO VV N = 315^a^	*p*-value
*Primary outcome*										
1-year post-transplant mortality	905	230 (25%)	53 (33%)	177 (24%)	**0.014**^b^	340	96 (28%)	53 (33%)	43 (24%)	0.059^b^

*Secondary outcomes*										
Removal from the waiting list for deterioration/death	1,551	459 (30%)	129 (41%)	330 (27%)	**<0.001**^b^	630	222 (35%)	129 (41%)	93 (30%)	**0.003**^b^
30-day post-transplant mortality	1,027	78 (7.6%)	18 (10%)	60 (7.0%)	0.14^b^	378	37 (9.8%)	18 (10%)	19 (9.4%)	0.8^b^
90-day post-transplant mortality	1,016	142 (14%)	32 (18%)	110 (13%)	0.065^b^	372	59 (16%)	32 (18%)	27 (14%)	0.2^b^
5-year post-transplant mortality	256	161 (63%)	34 (56%)	127 (65%)	0.2^b^	118	72 (61%)	34 (56%)	38 (67%)	0.2^b^
Days from ECMO initiation to removal from the waiting list for deterioration/death	459	14 (7, 27)	13 (6, 22)	14 (8, 30)	**0.044**^c^	222	14 (7, 27)	13 (6, 22)	17 (10, 35)	**0.009**^c^
Transplantation rate	1,551	1,053 (68%)	179 (57%)	874 (71%)	**<0.001**^b^	630	390 (62%)	179 (57%)	211 (67%)	**0.009**^b^
Type of transplant	1,053				0.2^b^	390				0.14^b^
Double lung		987 (94%)	164 (92%)	823 (94%)			365 (94%)	164 (92%)	201 (95%)	
Single lung		66 (6.3%)	15 (8.4%)	51 (5.8%)			25 (6.4%)	15 (8.4%)	10 (4.7%)	
On ECMO at transplant	1,053	915 (87%)	154 (86%)	761 (87%)	0.7^b^	390	332 (85%)	154 (86%)	178 (84%)	0.6^b^
Days from ECMO initiation to transplant	1,053	11 (5, 36)	11 (5, 19)	12 (5, 45)	**0.036**^c^	390	11 (5, 24)	11 (5, 19)	12 (5, 35)	0.2^c^
Ventilator support post-transplant	1,027	1,016 (99%)	175 (100%)	841 (99%)	0.2^d^	378	375 (99%)	175 (100%)	200 (99%)	0.3^d^
ECMO at 72 hours post-transplant	1,024	319 (31%)	67 (38%)	252 (30%)	**0.025**^b^	377	140 (37%)	67 (38%)	73 (36%)	0.7^b^
Length of stay post-transplant	996	32 (20, 55)	33 (20, 53)	32 (20, 55)	0.7^c^	366	34 (20, 61)	33 (20, 53)	35 (20, 66)	0.2^c^
Graft failure	1,023	875 (86%)	144 (83%)	731 (86%)	0.3^b^	376	316 (84%)	144 (83%)	172 (85%)	0.7^b^
Graft survival time (days)	1,029	707 (245, 1,122)	679 (181, 1461)	709 (260, 1101)	>0.9^c^	380	698 (186, 1,429)	679 (181, 1,461)	707 (202, 1,223)	>0.9^c^

Abbreviations: ECMO, extracorporeal membrane oxygenation; IQR, interquartile range; VA, venoarterial; VV, venovenous.

Bold value represents *p*<0.05.

a Median (IQR) or frequency (%).

b Pearson's chi-square test.

c Wilcoxon rank-sum test.

d Fisher's exact test.

In the Cox-proportional hazard multivariate analysis, the risk of 1-year mortality with VA ECMO compared to VV ECMO was not statistically significant in both the overall (adjusted hazard ratio [aHR] 1.01, 95% confidence interval [CI] 0.65-1.60, *p* > 0.9) and matched cohort (aHR 1.24, 95% CI 0.78-1.97, *p* = 0.4) (Table 3). The risk of 1-year mortality was not statistically significant with each increase in PVR (aHR 0.98, 95% CI 0.94-1.03, *p* = 0.4 in overall cohort; aHR = 0.96, 95% CI 0.91-1.01, *p* = 0.077 in matched cohort) or PAPI (aHR 0.98, 95% CI 0.96-1.01 in overall cohort; aHR = 0.97, 95% CI 0.94-1.01, *p* = 0.087 in the matched cohort) (Table S1).

**Table 3:** Cox-Proportional Hazard Ratio for 1-Year Mortality and Subgroup Analysis of Patients With ILD Listed for Lung Transplantation With VA ECMO Compared to VV ECMO

	Overall cohort			Matched cohort		
	*n*	aHR (95% CI)	*p*-value	*n*	aHR (95% CI)	*p*-value
**Primary outcome** *1-year post-transplant mortality*	1,055	1.01 (0.65-1.60)	>0.9	390	1.24 (0.78-1.97)	0.4
						
**Subgroup analysis**						
*PVR* (WU)						
<3	534	1.47 (0.75-2.84)	0.257	130	0.82 (0.41-1.66)	0.598
3-9	455	1.79 (0.95-3.08)	0.073	203	1.11 (0.58-2.11)	0.743
>9	66	1.67 (0.29-9.49)	0.561	57	2.73 (0.277-26.9)	0.390
*ECMO era by year of transplant*						
≤2019	361	1.08 (0.55-2.10)	0.82	159	0.83 (0.37-1.89)	0.665
2019-2022	333	1.70 (0.82-3.51)	0.149	107	1.73 (0.69-4.30)	0.235
≥2022	361	1.30 (0.70-2.40)	0. 405	124	1.72 (0.55-5.31)	0.348
*Age*						
<50	429	1.43 (0.74-2.76)	0.278	163	0.82 (0.37-1.78)	0.609
≥50	626	1.43 (0.85-2.38)	0.169	227	1.89 (0.96-3.70)	0.064
*Diagnosis*						
IPF	372	1.21 (0.58-2.51)	0.606	143	0.99 (0.39-2.55)	0.997
CTD-ILD	71	1.63 (0.04-54.7)	0.786	39	8.57 (0.133-551)	0.312
COVID	205	**7.12 (1.90-26.6)**	**0.003**	43	**20 (1.61-259)**	**0.020**
*Ambulation status*						
Ambulatory	483	1.63 (0.87-3.01)	0.121	165	1.89 (0.81-4.35)	0.137
Nonambulatory	572	1.36 (0.90-2.04)	0.140	225	1.02 (0.54-1.92)	0.945
*ECMO cannulation approach*						
Central	464	1.44 (0.86-2.40)	0.159	218	1.03 (0.56-1.89)	0.926
Peripheral	591	1.51 (0.81-2.82)	0.193	172	2.15 (0.87-5.28)	0.096
*Unimputed dataset*	1055	1.02 (0.60-1.73)	>0.9	391	0.87 (0.46-1.62)	0.7

Abbreviations: aHR, adjusted hazard ratio; CI, confidence interval; CTD-ILD, connective tissue disease–related interstitial lung disease; ECMO, extracorporeal membrane oxygenation; IPF, idiopathic pulmonary fibrosis; PVR, pulmonary vascular resistance; VA, venoarterial; VV, venovenous; WU, Woods Unit.

Bold value represents *p*<0.05.

**Figure 1:**
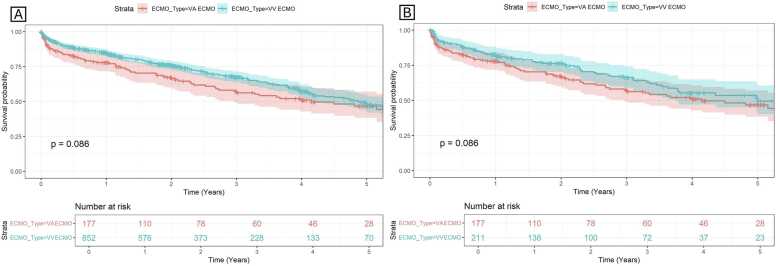
Kaplan-Meier survival curves comparing ILD patients bridged with venoarterial (VA) vs venovenous (VV) ECMO: (A) the overall cohort and (B) the matched cohort, with the log-rank test *p*-value displayed in each panel. ILD, interstitial lung disease; ECMO, extracorporeal membrane oxygenation.

The subgroup analysis showed a higher risk of 1-year mortality with VA compared to VV ECMO for COVID pulmonary fibrosis in both overall (aHR 7.12, 95% CI 1.90-26.6, *p* = 0.003) and the matched (aHR 20, 95% CI 1.61-259, *p* = 0.020) cohorts. The trends of the 1-year mortality with VA vs VV ECMO otherwise remained unchanged when subgrouped by PVR, ECMO era by transplant year, age, ambulation status, ECMO cannulation approach, and with the unimputed dataset (Table 3). The Kaplan-Meier survival curves up to 5 years post-transplant were similar with VA ECMO, when compared to VV ECMO, in both the overall and matched cohort (*p* = 0.086) (Figure 1).

## Discussion

In this retrospective UNOS cohort study, we evaluated the outcomes of VA vs VV ECMO as a bridge to LTx in patients with ILD. The post-transplant outcomes were similar in patients bridged with VA vs VV ECMO. However, the rate of delisting due to clinical deterioration or death was higher for VA compared to VV ECMO in both the overall and matched cohorts. Subgroup analysis showed a higher 1-year mortality with VA ECMO in the setting of COVID pulmonary fibrosis.

Patients with fibrotic ILD, including IPF and non-IPF etiologies such as fibrotic nonspecific interstitial pneumonia, and fibrotic hypersensitivity pneumonitis have an overall prognosis of 3 to 5 years of survival without a LTx.^12^, ^13^ ILD is often times associated with PH secondary to endothelial dysfunction, vascular remodeling, proliferative changes in the pulmonary vascular cells, and degenerative alterations in the lung parenchyma.^14^ The presence of concomitant PH and RV dysfunction portends worse outcomes with 2- to 5-fold increased risk of death.^15^ The consensus guidelines from the International Society for Heart and Lung Transplant recommend that ILD with any degree of PH as determined by right heart catheterization or 2D echocardiography be listed for LTx.^16^ Assessment of the severity of PH and its consequences based on PAP as measured by right heart catheterization or echocardiography could be sometimes misleading as studies on explants have shown that pulmonary arterial vasculopathy is seen in patients with fibrotic ILD irrespective of the presence and/or severity of PH.^17^ A retrospective study showed the development of new-onset RV dysfunction in 18% of the cohort following VV ECMO support, with higher ECMO flow rates and longer duration of support correlating with worsening RV function.^18^ Thus, theoretically, VA ECMO may offer an advantage over VV ECMO in the setting of RV dysfunction as it unloads both ventricles and bypasses the vasculopathic pulmonary circulation characteristic of PH.

Our study, however, showed that VA ECMO had a higher delisting from the waitlist due to death or deterioration compared to VV ECMO in our study. The reasons are likely multifactorial with the most significant being that patients with greater severity of illness are more likely to be initiated on VA ECMO. However, the retrospective design of our study, based on the UNOS STAR database, limits the ability to capture the nuanced clinical judgments made by treating teams that influence the selection between VA vs VV ECMO. VA ECMO inherently presents additional risks due to arterial cannulation, including limb ischemia, increased incidence of acute kidney injury, higher rates of hemolysis, neurological complications, and the risk of North-South (Harlequin) syndrome with peripheral cannulation.^6^, ^19^ The limited set of variables available in the database—including the absence of data on nonpulmonary organ dysfunction, ECMO-parameters such as flow rate, and ECMO-related complications—reduces the granularity of the data and hinders a more comprehensive understanding of the factors contributing to differences in successful bridging to LTx. However, once successfully bridged to transplant, the adjusted post-transplant outcomes at 1 year were similar in patients bridged with VA or VV ECMO in the matched cohort.

Studies comparing the outcomes of VA vs VV ECMO in the setting of ILD are limited in the existing literature. Chicotka et al demonstrated increased survival to transplantation with VA compared to VV ECMO in their cohort of 50 patients with ILD and PH.^9^ This contrasts with our study, where we noted a decreased survival to transplantation with VA ECMO. In their study, the mean PAP for patients on VA and VV ECMO were 82 and 56 mm Hg compared to 34 and 28 mm Hg in our cohort, respectively. This comparison is limited, however, by the uncertainty regarding whether the invasive hemodynamic data were obtained while the patient was on or off ECMO support, as values measured during ECMO may be misleading due to circuit-related alterations in hemodynamics. A previously published study on the UNOS database, including patients between 2015 and 2020, showed a hazard ratio of death or removal pretransplant of 1.33 (1.02-1.75) in patients with VA ECMO compared to VV ECMO.^7^ This finding was similar to our results. Among ILD patients on ECMO who are awake or ambulating, a meta-analysis showed that ambulatory VA ECMO had better in-hospital mortality than ambulatory VV ECMO. This finding was, however, reported with a very-low GRADE certainty due to reliance on 2 small retrospective studies with sample sizes of 25 and 16 patients.^10^ In contrast, our study showed no difference in 1-year post-transplant mortality with ambulatory VA or VV ECMO in the matched cohort.

Studies have identified PVR as an early predictor of mortality in patients with fibrotic ILD, with outcomes showing no significant correlation with other invasive or noninvasive hemodynamic parameters.^20^, ^21^ This may be attributed to the fact that PAP often declines in the late stages of decompensated RV failure. In contrast, PVR—calculated using PAP, PCWP, and CO, provides a more accurate reflection of the hemodynamic burden of PH and RV failure. PAPI, a novel hemodynamic index that represents the interaction between the RV and pulmonary artery and provides insight into the ability of the RV to generate pulsatile flow, is associated with RV function and survival in patients with PH.^11^ Our study did not show an association of PVR or PAPI with the hazard of 1-year mortality. One potential explanation is the lack of timing information for hemodynamic measurements in the database, as many values may have been recorded before clinical decompensation.

Although post-transplant outcomes for COVID-19–related pulmonary fibrosis are comparable to those with pulmonary fibrosis from other causes, our subgroup analysis revealed higher 1-year post-transplant mortality among patients with COVID-19 pulmonary fibrosis bridged with VA- compared to VV ECMO.^22^, ^23^ The observed finding may be attributed to the heightened inflammatory response associated with COVID-19, which can increase the vascular complications such as thromboembolism, particularly with arterial cannulation in VA ECMO. Additionally, the inherent characteristics of the patient population may contribute, as individuals with COVID-19 are more likely to require VA ECMO due to postcardiac arrest or myocardial dysfunction.^24^ This observation may suggest that candidates with COVID-19 requiring VA ECMO support should be carefully reconsidered before proceeding with LTx, given the elevated risk of post-transplant mortality.

The strengths of our study include a large sample size, which addresses a common limitation in ECMO-related research, and the use of 1:1 propensity score matching to mitigate selection bias between groups. Additionally, we strengthened our analysis by evaluating the influence of pulmonary hemodynamics on outcomes and conducting subgroup analyses across distinct cohort groups.

Limitations of this study include its retrospective design and reliance on the UNOS STAR database, which captures a limited set of variables that may not fully reflect the clinical complexity of individual patients, introducing the potential for unknown confounders. For instance, the criteria guiding the choice between VA and VV ECMO cannulation are not clearly defined and may inherently influence outcomes. Additional missing data posed additional challenges: while imputation was performed for hemodynamic variables, missing post-transplant outcome data for 24 patients could not be imputed, potentially impacting the robustness of the analysis. The timing of hemodynamic measurements relative to ECMO cannulation is also uncertain; values recorded while on ECMO may be inaccurate due to circuit-related alterations, whereas data collected well before listing may not represent the patient’s current physiological status. Furthermore, the database lacks information on important pretransplant complications, such as the right or left ventricular dysfunction, development of limb ischemia, serial lactate trends particularly during ECMO support, limiting our ability to investigate mechanisms underlying the poor outcomes observed with VA. Also, data on peripheral VA ECMO with femoral arterial return vs central VA ECMO or hybrid RV-PA cannulation were unavailable, which could also affect the outcomes. Lastly, donor-related variables, which may confound post-transplant outcomes, were not included to streamline the analysis.

## Conclusion

In this retrospective study of the UNOS STAR database spanning many years, post lung transplant outcomes were comparable between patients with ILD bridged with VA or VV ECMO after adjusting for confounders. However, VV ECMO was associated with a higher rate of successful bridging to LTx compared to VA ECMO. Given the limited granularity of available clinical data, prospective randomized controlled studies are needed to elucidate outcome differences in ILD patients bridged with VA vs VV ECMO.

## CRediT authorship contribution statement

**Prasanth Balasubramanian:** conceptualization, methodology, formal analysis, writing and editing. **Pablo Moreno Franco:** writing – review and editing, data curation. **Sanjay Chaudhary:** writing, resources. **Francisco G. Alvarez:** writing – review, resources. **Maher Baz:** writing – review, resources. **Tathagat Narula:** writing – review, resources. **Sadia Z. Shah:** writing –review, resources. Mohammad Alomari: writing – review, resources. **Rohan Bongu:** writing –review, resources. **Anirban Bhattacharrya:** writing – review, resources. **Devang Sanghavi:** writing – review, resources. **Sean Kiley:** writing – review, resources. **Archer K. Martin:** writing – review, resources. **Nikki L. Matos:** writing – review, resources. **John C. Haney:** writing –review, resources. **Ian Makey:** writing – review, resources. **Mathew Thomas:** writing – review, resources. **Pramod K. Guru:** writing – review, resources. **Remzi Bag:** writing – conceptualization, writing – review, resources, supervision.

## Disclosure statement

The authors declare that they have no known competing financial interests or personal relationships that could have appeared to influence the work reported in this paper.

Acknowledgments and Funding: None.

## Footnotes

Appendix A Supplementary data associated with this article can be found in the online version at doi:10.1016/j.jhlto.2025.100287.
