# Supplementary material for: Outcomes with venoarterial vs venovenous extracorporeal membrane oxygenation as a bridge to lung transplantation in interstitial lung disease
Source: JHLT Open. 2025 May 13;9:100287. doi: 10.1016/j.jhlto.2025.100287 (PMC12166393; doi:10.1016/j.jhlto.2025.100287)
Supplement: Supplementary file 1 — Supplementary material [file mmc1.docx]

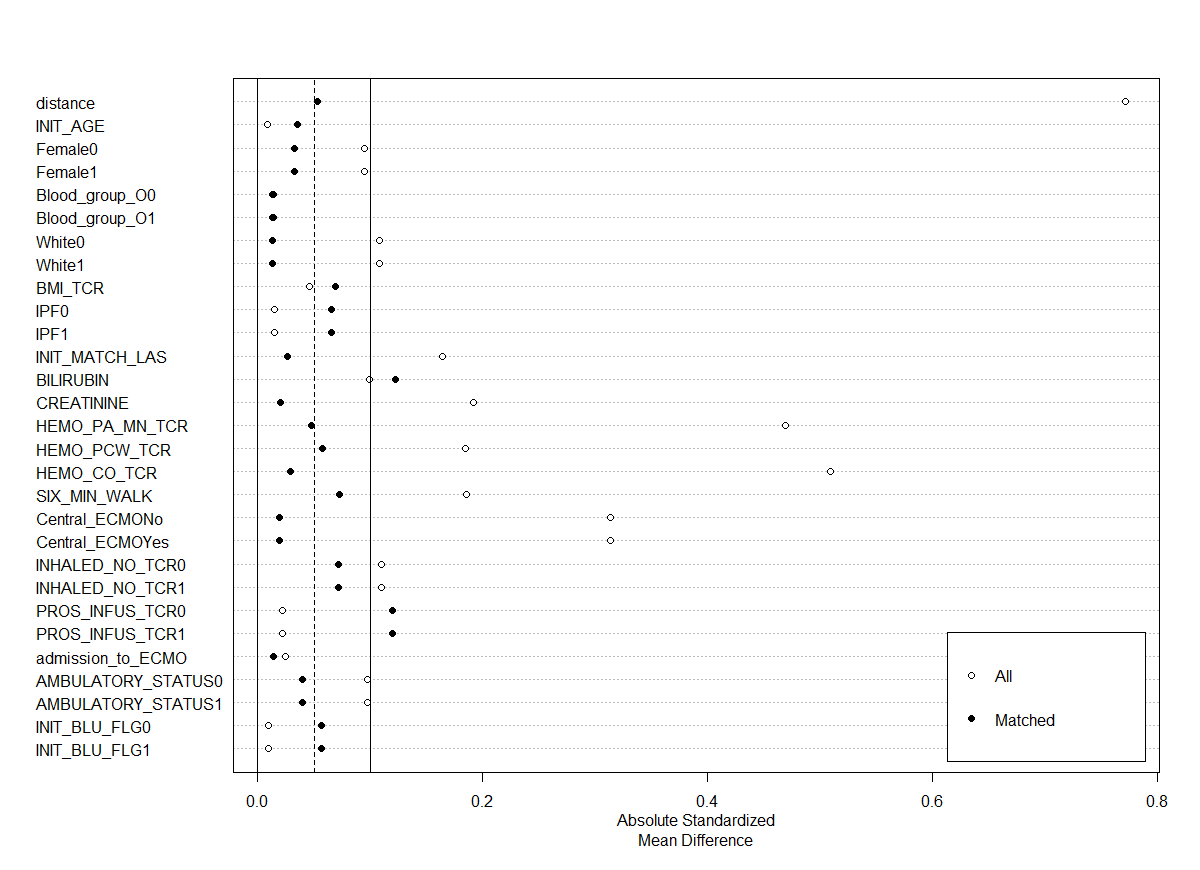


**Figure S1:** Summary plot of absolute standardized mean difference before and after propensity matching. TCR – data at listing, HEMO_PA_MN_TCR – Mean PA pressure at listing, HEMO_PCW_TCR – Pulmonary capillary wedge pressure at listing, HEMO_CO_TCR – cardiac output at listing, INIT_BLU_FLG – listed for bilateral lung transplant


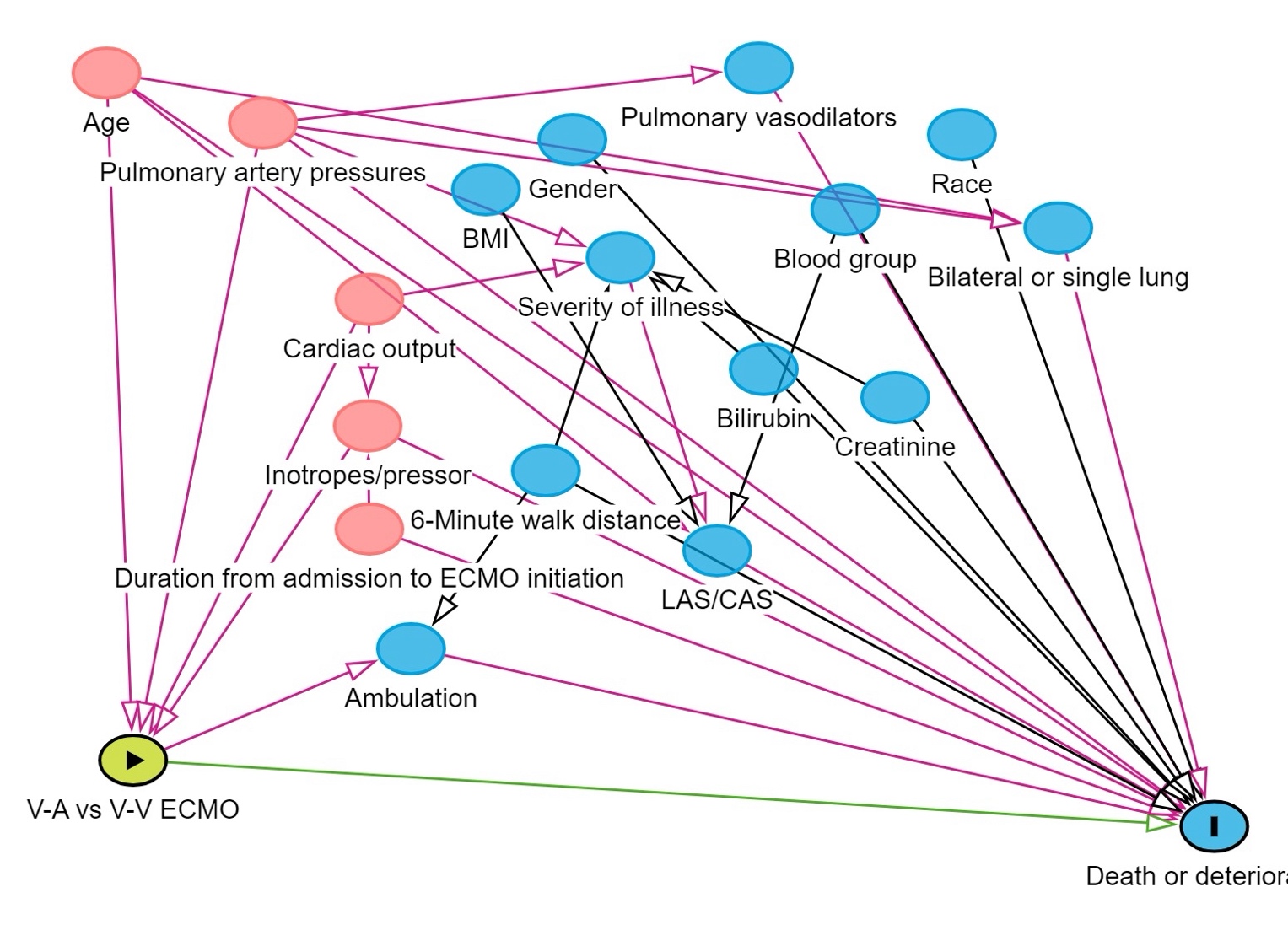


**Figure S2:** Directed acrylic graph showing the relationship between the exposure variable (type of ECMO) and outcome (death or deterioration) with other plausible variables and its paths as follows: Blue Circles = Ancestor of Outcome, Light Green Circle = Ancestor of Exposure, Light Red Circles = Ancestor of Exposure and Outcome, Green line = Causal Path, Maroon Line = Biasing Path.

Created using http://www.daggity.net

**Supplementary table 1: Multivariate cox-regressional analysis for 1-year post-transplant mortality**

|  | **Unmatched cohort** | | | **Matched cohort** | | |
| --- | --- | --- | --- | --- | --- | --- |
| **Characteristic** | **HR***^1^* | **95% CI***^1^* | **p-value** | **HR***^1^* | **95% CI***^1^* | **p-value** |
| **Age (years)** | 1.01 | 1.0, 1.03 | 0.2 | 0.99 | 0.97, 1.01 | 0.3 |
| **Diabetes** |  |  | 0.2 |  |  | 0.5 |
| No | — | — |  | — | — |  |
| Yes | 1.29 | 0.87, 1.91 |  | 1.19 | 0.69, 2.03 |  |
| **Female Sex** |  |  | **<0.001** |  |  | **0.033** |
| No | — | — |  | — | — |  |
| Yes | 2.02 | 1.36, 3.01 |  | 1.78 | 1.05, 3.03 |  |
| **Blood group O** |  |  | 0.7 |  |  | 0.7 |
| No | — | — |  | — | — |  |
| Yes | 0.94 | 0.66, 1.34 |  | 1.10 | 0.68, 1.79 |  |
| **White Race** |  |  | 0.6 |  |  | 0.073 |
| No | — | — |  | — | — |  |
| Yes | 1.11 | 0.75, 1.62 |  | 1.58 | 0.95, 2.61 |  |
| **Body Mass Index** | 1.00 | 0.97, 1.04 | 0.9 | 1.03 | 0.98, 1.08 | 0.3 |
| **Idiopahtic Pulmonary Fibrosis** |  |  | 0.3 |  |  | 0.7 |
| No | — | — |  | — | — |  |
| Yes | 0.81 | 0.54, 1.21 |  | 0.89 | 0.51, 1.58 |  |
| **LAS at listing** | 1.00 | 0.99, 1.01 | 0.8 | 1.00 | 0.99, 1.02 | 0.6 |
| **Bilirubin at registration** | 1.23 | 1.08, 1.40 | **0.015** | 1.15 | 0.86, 1.55 | 0.4 |
| **Creatinine at registration** | 2.32 | 1.43, 3.76 | **0.002** | 1.77 | 1.07, 2.93 | **0.047** |
| **6-mintue Walk Distance** | 1.00 | 1.00, 1.00 | >0.9 | 1.00 | 1.00, 1.00 | >0.9 |
| **PVR** | 0.98 | 0.94, 1.03 | 0.4 | 0.96 | 0.91, 1.01 | 0.077 |
| **PAPI** | 0.98 | 0.96, 1.01 | 0.2 | 0.97 | 0.94, 1.01 | 0.087 |
| **Inotropes at registration** |  |  | 0.7 |  |  |  |
| No | — | — |  |  |  |  |
| Yes | 0.84 | 0.35, 1.99 |  |  |  |  |
| **Inhaled NO at registration** |  |  | >0.9 |  |  | 0.6 |
| No | — | — |  | — | — |  |
| Yes | 0.96 | 0.38, 2.41 |  | 0.73 | 0.22, 2.39 |  |
| **Prostacyclin infusion at registration** |  |  | 0.6 |  |  | 0.14 |
| No | — | — |  | — | — |  |
| Yes | 0.63 | 0.08, 4.88 |  | 0.00 | 0.00, Inf |  |
| **VA ECMO** |  |  | >0.9 |  |  | 0.4 |
| No | — | — |  | — | — |  |
| Yes | 1.01 | 0.65, 1.60 |  | 1.24 | 0.78, 1.97 |  |
| **Central ECMO** |  |  | 0.13 |  |  | 0.052 |
| No | — | — |  | — | — |  |
| Yes | 1.32 | 0.93, 1.88 |  | 1.62 | 0.99, 2.68 |  |
| **Days from admission to ECMO initiation** | 1.00 | 0.99, 1.01 | >0.9 | 1.00 | 0.99, 1.01 | >0.9 |
| **Ambulatory status** |  |  | 0.4 |  |  | 0.2 |
| No | — | — |  | — | — |  |
| Yes | 0.85 | 0.59, 1.22 |  | 0.72 | 0.45, 1.17 |  |
| *^1^*HR = Hazard Ratio, CI = Confidence Interval | | | | | | |
| LAS – Lung allocation score, PVR-pulmonary vascular resistance, PAPI- pulmonary artery pulsatility index, VA-Veno-arterial,  ECMO-extracorporeal membrane oxygenation | | | | | | |
